# Dense Array EEG Source Estimation in Neocortical Epilepsy

**DOI:** 10.3389/fneur.2013.00042

**Published:** 2013-05-13

**Authors:** Madoka Yamazaki, Don M. Tucker, Marie Terrill, Ayataka Fujimoto, Takamichi Yamamoto

**Affiliations:** ^1^Department of Health Science, Daito Bunka UniversitySaitama, Japan; ^2^Comprehensive Epilepsy Center, Seirei Hamamatsu General HospitalShizuoka, Japan; ^3^Department of Psychology, University of OregonEugene, OR, USA; ^4^Electrical Geodesics, Inc.Eugene, OR, USA

**Keywords:** dense array EEG, source estimation, neocortical epilepsy, interictal spike, intracranial EEG

## Abstract

**Rationale:** Dense array EEG (dEEG) evenly covers the whole head surface with over 100 channels contributing to more accurate electrical source imaging due to the higher spatial and temporal resolution. Several studies have shown the clinical utility of dEEG in presurgical clinical evaluation of epilepsy. However validation studies measuring the accuracy of dEEG source imaging are still needed. This can be achieved through simultaneously recording both scalp dEEG with intracranial electrodes (icEEG), which is considered as the true measure of cortical activity at the source. The purpose of this study is to evaluate the accuracy of 256-channel dEEG electrical source estimation for interictal spikes.

**Methods:** Four patients with medically refractory neocortical epilepsy, all surgical candidates, underwent subdural electrode implantation to determine ictal onset and define functional areas. One patient showed a lesion on the magnetic resonance imaging in the right parietal lobe. The patient underwent simultaneous recording of interictal spikes by both scalp 256-channelsvdEEG and icEEG. The dEEG was used to non-invasively estimate the source of the interictal spikes detected by the 256-channel dEEG array, which was then compared to the activity measured directly at the source by the icEEG.

**Results:** From the four patients, a total of 287 interictal spikes were measured with the icEEG. One hundred fifty-five of the 287 spikes (54%) were visually detected by the dEEG upon examination of the 256 channel head surface array. The spike amplitudes detected by the 256-channel dEEG correlated with icEEG spike amplitudes (*p* < 0.01). All spikes detected in dEEG were localized to the same lobe correctly.

**Conclusion:** Our study demonstrates that 256-channel dEEG can reliably detect interictal spikes and localize them with reasonable accuracy. Two hundred fifty-six-channel dEEG may be clinically useful in the presurgical workup for epilepsy and also reduce the need for invasive EEG evaluation.

## Introduction

For patients with intractable epilepsy, surgical therapy is an important treatment option. Although the propagation of seizures may involve complex cerebral networks, surgical resection of the cortical zone of seizure onset may be effective, if this zone can be identified and delineated from surrounding tissue. The first stage of diagnostic tests is typically non-invasive, with the goal being to characterize the epileptogenic zone as comprehensively as possible in order to guide the next stage of invasive testing with intracranial electrodes that confirms the decision on resective surgery.

Magnetoencephalography (MEG) and electroencephalography (EEG) have both been used as non-invasive methods in the electrophysiological evaluation of the epileptogenic zone. A number of studies has shown that multi-channel MEG is useful for dipole localization of interictal spike events, especially in neocortical epilepsy, where the sources may be more superficial (closer to the skull) compared to epilepsy with deeper sources, such as mesial temporal lobe epilepsy (Ricci et al., 1987; Gallen et al., 1995; Knowlton et al., 1997; Stefan et al., 2003; Huiskamp et al., 2010). MEG is inherently insensitive to deeper brain sources because magnetic signals fall off by the square of the distance from the source. Although EEG has better depth sensitivity than MEG, and it is sensitive to radial (gyral) as well as tangential (sulcal) sources, the electrical volume conduction of EEG is distorted by the resistive skull, requiring detailed computational modeling for an accurate inverse estimation (Ebersole, 2000; Vanrumste et al., 2000; Baumgartner, 2004; Plummer et al., 2007).

Although accurate electrical head modeling is now available for EEG, based on the patient’s magnetic resonance imaging (MRI) and CT (for skull conductivity), the conventional 21-channel scalp EEG does not have high enough spatial resolution to allow for accurate source localization in most cases (Tucker et al., 2004; Holmes et al., 2005). Adequate source localization requires adequate spatial sampling in addition to sophisticated source analysis techniques. Freeman et al. (2003) reported that the spatial Nyquist of the human scalp EEG necessitates inter-sensor distances of <10 mm. Recently it has become possible to record dense array EEG (dEEG) of up to 256 channels, providing 20–25 mm interelectrode distances for most adult head sizes. Several studies have shown that interictal source localization with dEEG suggests its usefulness in neurosurgical planning in epilepsy (Lantz et al., 2003a,b; Michel et al., 2004a; Holmes, 2008). However validation studies measuring the accuracy of dEEG source imaging are still needed. This can be achieved through simultaneously recording the scalp dEEG with intracranial electrodes (icEEG), which provides a local measure of electrical activity at the gyral cortical (pial) surface. In previous studies we have investigated the sensitivity and accuracy of dEEG source analysis comparing 256-channel dEEG source analysis of interictal activity and simultaneously recorded icEEG in patients with mesial temporal lobe epilepsy (Yamazaki et al., 2012a,b). In the present study, we extend this approach to investigate the accuracy of source estimation with dEEG in patients with neocortical epilepsy using simultaneous dEEG and icEEG recording.

## Materials and Methods

### Patients

We studied four patients, each of whom had suffered from intractable localization related epilepsy for at least 2 years. Each patient was a surgical candidate and underwent a presurgical workup including conventional long-term EEG monitoring, MRI, 125-Iomazenil (IMZ)-single-photon emission tomography (SPECT), 18-F fluorodeoxyglucose (FDG)-Positron emission tomography (PET), and neuropsychological testing. Clinical information for these patients is summarized in Table 1. Case 1 had right parietal cortical dysplasia.

**Table 1:** **Case summary**.

	Case 1	Case 2	Case 3	Case 4
Age/sex	28 year/F	38 year/M	27 year/M	21 year/F
Onset	3 year	8 year	5 year	17 year
Sz type	SPS, CPS, sGTC	SPS, CPS, sGTC	SPS, sGTC	CPS, sGTC
MRI	R-P cortical dysplasia	Normal	Normal	Normal
IMZ-SPECT*	R-P	L-F, mT, laT	Bilateral-F, P	R mT
FDG-PET**	R C-P	L-F, mT, laT	R-P	R mT, laT
EEG Sz onset	C4, P4	Fp1, F7, T3	P4, T4	T4, T6
Interictal	C4, P4	Fp1, F7, T3	C4, P4, T6	Fp2, F8, T4
Subdural electrodes location	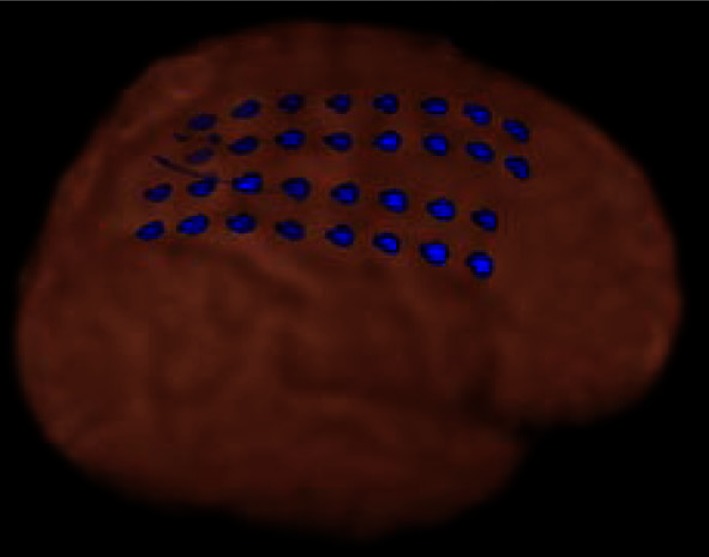	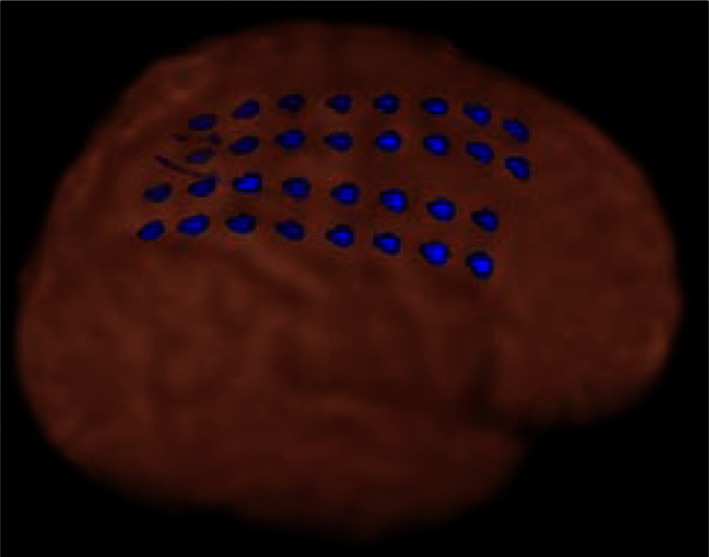	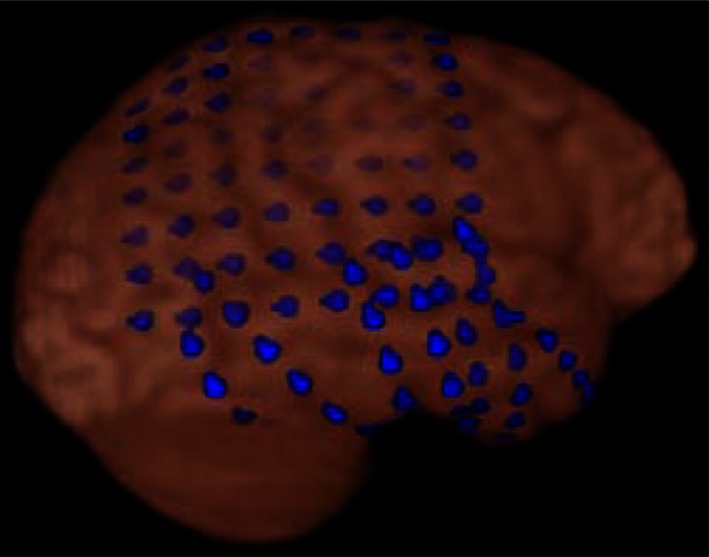	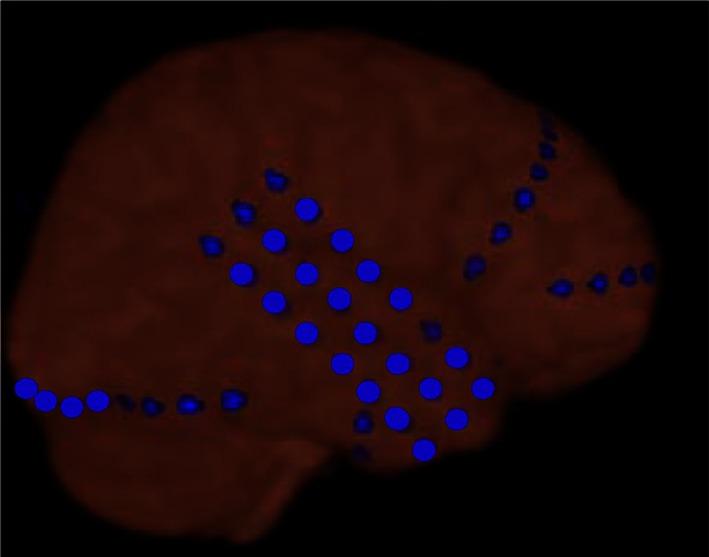

SPS, simple partial seizure; CPS, complex partial seizure; sGTC, secondary generalized tonic clonic seizure; *, hypoperfusion area; **, hypometabolism area; mT, mesial temporal lobe; laT, lateral temporal lobe.

We received approval for this study from Seirei Hamamatsu General Hospital Human Subject Committee and informed consent was obtained from all patients.

### Intracranial EEG recording

Subdural strip and grid electrodes (Unique medical, Tokyo, Japan) were implanted for each patient in order to delineate the epileptogenic zone for cortical excision, and to separate it from functional areas. All electrode contacts were platinum, and interelectrode distance was 10 mm. The location and types of subdural electrodes used for each patient are shown in Table 1.

### Dense array EEG recording

The dEEG was recorded with the 256-channel Geodesic Sensor Net (Electrical Geodesics, Inc., Eugene, OR, USA), providing 257 electrodes (including vertex reference) covering the face and neck as well as cranium, with 20–25 mm interelectrode distances. The coverage of the face and neck is important for measuring the downward projection of electrical potentials from basal brain regions.

### Simultaneous icEEG and dEEG acquisition

We conducted simultaneous icEEG recording with NicoletOne (CareFusion, Middleton, WI, USA) and dEEG with Net Amp300 (Electrical Geodesics, Inc., Eugene, OR, USA) at 1 kHz sampling with bandpass filter 0.1 and 400 Hz for approximately 30–40 min. A digital pulse from the icEEG system was provided to the dEEG acquisition system for synchronization. Prior to the simultaneous icEEG-dEEG recording, each patient recovered from the intracranial placement for at least 3 days, allowing surgical wounds to heal to avoid infection risk during simultaneous scalp recording. There were no complications due to any of the simultaneous recordings.

### Data analysis

Frequent epileptiform discharges were selected during artifact free periods in the simultaneous dEEG and icEEG recordings. Interictal spikes seen in icEEG were visually identified by a certified EEG technologist (MY) and confirmed by a board-certified clinical epileptologist (AF). As expected, many of the icEEG spikes were not detectible by inspection of the dEEG. We calculated the spike detection rate of 256-channel dEEG (with the icEEG detection as the criterion), and we measured the average maximum amplitude for both the icEEG detected spikes and the smaller subset of dEEG detected spikes.

Electrical source localization was conducted at the rising phase for the dEEG detected spikes with a linear inverse method (LAURA) using the GeoSource 1.0 software package^1^ within the space of a 3D head model derived from the Montreal Neurological Institute’s average adult MRI. The LAURA constraint provides results very similar to the low-resolution electromagnetic tomography (LORETA) spatial Laplacian constraint (Pascual-Marqui et al., 2002), and this smoothing constraint has been shown to provide stable source estimation of interictal epileptiform events in neurosurgical planning for epilepsy (Lantz et al., 2003a,b; Michel et al., 2004a,b).

## Results

### Spike detection rate

A total of 287 spikes were recorded in icEEG with four patients during recordings of approximately 30–40 min each. One hundred fifty-five of these spikes (54%) were also clearly distinguishable from background activity in the simultaneously recorded dEEG. The dEEG detection rate for the spikes verified by icEEG for each patient is summarized in Table 2. Case 2 showed two independent patterns of interictal spikes, one originating from the left frontal region and the other from the left mesial temporal region. The detection rate of neocortical spikes was 56% and that for mesial temporal spikes in this case was 39%.

**Table 2:** **Spike detection rate**.

Spike location		Case 1	Case 2		Case 3	Case 4
		Parietal lobe	Mesial temporal lobe	Frontal lobe	Parietal lobe	Lateral temporal lobe
No. of spilkes	Detectable	40	14	44	36	21
	Undetectable	31	22	39	20	20
	Detection rate (%)	56	39	53	64	51
Amplitude (μV)	Detectable	907	1112	902	989	1075
	Undetectable	686	763	762	755	823

### icEEG amplitude of detectable spikes

The average maximal icEEG amplitude of neocortical interictal spikes that were also detectable by the scalp dEEG was 968 μV (standard deviation of 139 μV), significantly higher (*p* < 0.01) than that of dEEG undetectable spikes (757 μV; standard deviation of 102 μV). The average maximal icEEG amplitude of mesial temporal spikes that were also detectable by the scalp dEEG was 1112 μV (standard deviation of 197 μV), significantly higher (*p* < 0.01) than that of dEEG undetectable spikes (763 μV; standard deviation of 172 μV).

### Dense array EEG source estimation

**Figure 1:**
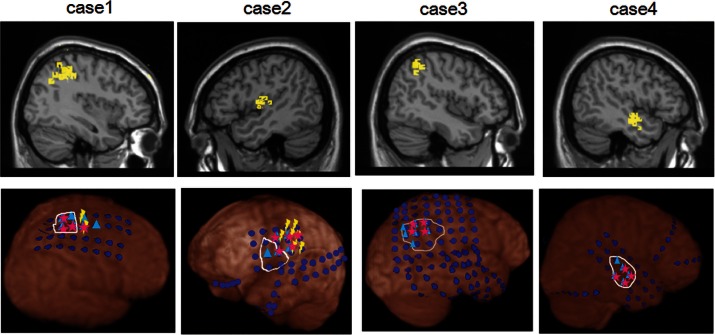
**dEEG source estimation**. Upper: dEEG source estimation by dEEG superimposed on a standard MRI, down: icEEG findings, 
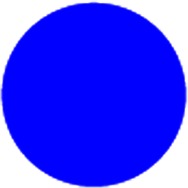
: subdural electrode, 
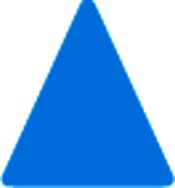
: interictal discharges, 
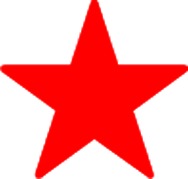
: ictal onset, white circle: resected area, 
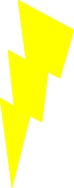
: eloquent area

Figure 1 shows dEEG source estimation for the dEEG detected icEEG spikes in each patient, visualized by displaying the voxel with maximal source amplitude (as well as voxels with similar amplitudes) using a section from the MNI typical brain image^2^ consistent with the MNI atlas used for the cortical dipole locations in the GeoSource software. All spikes detected in dEEG were localized to the same lobe correctly and 141 of 155 spikes (91%) were well localized, close to the position confirmed by subdural electrodes.

**Figure 2:**
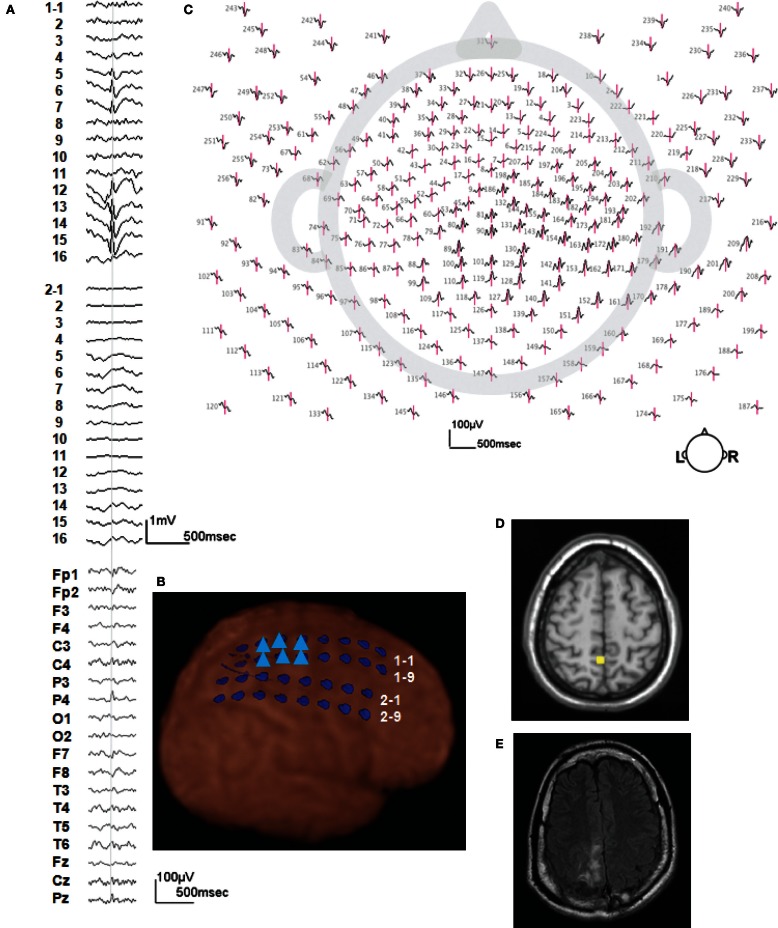
**A typical example of icEEG and dEEG source estimation (case 1)**. **(A)** The icEEG (upper) shows a right parietal spike. Interictal spike is shown at electrodes # 1–5–7 and 12–16 which are located over the parietal region. The EEG (lower) is simultaneous recorded 256-channel dEEG with 19-channel of 10/20 display. **(B)** Placement of subdural electrodes and the location of the interictal spike. ▲ Indicates the electrodes which show the interictal spike. **(C)** Two hundred fifty-six-channel dEEG topographic plot of the corresponding spike. The view is looking down on top of the head with nose at the top. The 256-channnel topographic plot was instructive in localizing the spike to the right parietal lobe. **(D)** The source estimation by dEEG superimposed on a standard MRI. The interictal spike is localized to right parietal head region. **(E)** Post-operative MRI.

Figure 2 shows more detail for a typical example of icEEG and dEEG source estimation. For this selected spike, the icEEG and conventional EEG recordings are shown in Figure 2A. The locations of the icEEG strips are shown in Figure 2B. The head surface (scalp, face, and neck) distribution of the dEEG potentials is shown in Figure 2C. From inspection, this dEEG head surface topography is consistent with a mostly radial source in the right posterior midline, with diffuse inversions over both sides of the face indicating the approximate radial source orientation. The peak of the non-invasive dEEG source estimation for this spike is shown in Figure 2D consistent with visual inspection of the surface array, and the post-operative MRI shows the resected region in Figure 2E.

### Surgical outcome

All patients underwent resective surgery based on the intracranial icEEG findings, functional mapping, and imaging studies. Pathological findings are case 1: FCD type IIB, case 2: FCD type IIB, case 3: FCD type IA, and case 4: no abnormality. Surgical outcome is Engel class Ia (case 3, 4), IId (case 1), and IIIa (case 2) within the post-operative follow-up period 23–35 months at this time (mean, 30 months).

## Discussion

Simultaneous recording of non-invasive dEEG with icEEG allowed a direct comparison of the sensitivity of dEEG to epileptiform events (spikes) that were confirmed by intracranial recordings. In these four patients with neocortical (extra temporal) epilepsy, the non-invasive dEEG localization of spikes predicted not only the intracranial location of the spikes, but also the cortical location of seizure onset (Figure 1).

Clearly there were many spikes visible in the icEEG that were not detected through visual inspection of the dEEG. For the neocortical spikes identified by icEEG in the present study, the dEEG spike detection rate was 56%. As would be expected, the dEEG detected spikes were consistently larger in amplitude than the dEEG undetected spikes in each patient. For the mesial temporal spikes examined in our previous studies with simultaneous icEEG and dEEG (Yamazaki et al., 2012a,b), the dEEG detection rates were somewhat lower, 45 and 42%, respectively. Also as expected, to be detectable by visual inspection of the dEEG, the mesial temporal lobe spikes in those studies were typically larger in amplitude (averaging 1236 μV; Yamazaki et al., 2012b) compared with the dEEG detected neocortical spikes of the present study (averaging 968 μV). For the one patient of the present study with mesial temporal spikes, the 39% dEEG detection rate was similar to the previous rates for detecting mesial temporal spikes.

A higher non-invasive detection sensitivity to neocortical than mesial temporal spikes has also been observed in those MEG studies where validation has also been obtained from simultaneous icEEG recording. Compared with dEEG, MEG is particularly insensitive to the deep sources presented by mesial temporal lobe spikes. Huiskamp et al., 2010 reported that whole head MEG detected only 28% of the icEEG spikes generated in the mesial temporal lobe, whereas it detected 70% of the icEEG detected spikes from the lateral temporal lobe and extra temporal regions. Similarly, Oishi et al., 2002 observed that MEG detected only 26% of the mesial temporal spikes, but 53% of lateral frontal spikes that were confirmed by simultaneous icEEG recording. Considering our previous results and the present study, 256-channel dEEG is roughly equivalent to whole head MEG in detecting neocortical sites but superior to MEG in detecting mesial temporal lobe spikes.

Magnetic fields are less distorted by the skull, cerebral fluid, and scalp than electrical fields (Nakasato et al., 1994; Ebersole, 1999; Minassian et al., 1999; Otsubo et al., 1999; Morioka et al., 2000). However, the accuracy of MEG for deep sources, such as in the mesial temporal lobe, appears limited because magnetic signals fall off by the square of the distance (Mikuni et al., 1997; Oishi et al., 2002; Rampp and Stefan, 2007). In addition, the 256-channel dEEG sensor net includes sensors on the face and neck that improve the characterization of the electrical fields from the temporal lobe that are directed inferiorly and anteriorly, where even whole head MEG has limited coverage. Finally, the inclusion of accurate head conductivity information, such as from the MRI and CT atlas used for finite difference conductivity modeling in GeoSource software in the present analyses, is now helping to deal with the ambiguities of electrical volume conduction that plague EEG source localization.

Presurgical evaluation for what is typically called neocortical epilepsy, located outside the mesial temporal lobe (and archicortical hippocampus), requires precise accuracy because the epileptogenic zones are often located adjacent to critical functional areas of cortex (Otsubo et al., 1997; Minassian et al., 1999; Chitoku et al., 2001). For the four patients of the present study, the non-invasive dEEG source localization of the patient’s typical interictal spikes was predictive of the icEEG localization of not only the interictal spikes but also the seizure onset. Although the interictal events do not always localize to the same region as seizure onset, the utility of careful non-invasive localization of interictal events as a first stage in the presurgical workup is consistent with previous dEEG studies on electric source imaging in epilepsy (Michel et al., 2004b; Brodbeck et al., 2010). Nonetheless, non-invasive localization of seizure onset is an important confirmation for the hypotheses generated from localizing interictal events, and may be an important form of converging evidence in preparing for icEEG mapping of seizure onset (Holmes et al., 2008, 2010). Non-invasive 256-channel dEEG is now available for long-term monitoring for seizure onset; although more challenging than conventional EEG monitoring, it is more practical than attempting seizure monitoring with MEG.

There were several important technical limitations of this study that should be addressed in future research. We used standard 3D sensor positions, rather than the precise 3D locations of the 256-channels for each individual net application, such as can be obtained with photogrammetry (Russell et al., 2005). Even with careful placement of the net, the sensor positions can vary in relation to the cortex from patient to patient depending on head shape and the tissues of the face and neck. In addition, we used the GeoSource atlas finite difference model of head conductivity, based on the MNI brain template (and skull CT fit to that template), rather than building an electrical conductivity model based on the individual’s MRI and CT for the source estimation. Brodbeck et al., 2011 examined the sensitivity and specificity of dEEG source estimation comparing individual MRI and standard MRI template and showed the benefit of using individual MRI. For example, Case 1 in the present series had a focal cortical dysplasia in the right parietal region; rather than using the GeoSource atlas it would have been useful if we could compare the source localization of the spikes with the lesion location precisely by building the conductivity model from the patient’s MR and CT.

## Conclusion

Simultaneous recording of dEEG and icEEG allows direct examination of the accuracy of localizing cortical electrical sources from non-invasive dEEG recordings. In four patients with neocortical epilepsy examined with 256-channel dEEG, we observed good prediction of both the interictal spikes and seizure onset as verified by icEEG. Careful non-invasive analysis of the patient’s typical interictal events with dEEG may be an important first step in the neurosurgical planning for resection of the seizure onset zone.

## Conflict of Interest Statement

The authors declare that the research was conducted in the absence of any commercial or financial relationships that could be construed as a potential conflict of interest.

## Footnotes

^1^http://www.egi.com

^2^www.bic.mni.mcgill.ca
